# Tetraspanin CD9 Promotes the Invasive Phenotype of Human Fibrosarcoma Cells via Upregulation of Matrix Metalloproteinase-9

**DOI:** 10.1371/journal.pone.0067766

**Published:** 2013-06-28

**Authors:** Michael J. Herr, Jayaprakash Kotha, Nikolaus Hagedorn, Blake Smith, Lisa K. Jennings

**Affiliations:** 1 The Vascular Biology Center of Excellence and the Department of Internal Medicine, University of Tennessee Health Science Center, Memphis, Tennessee, United States of America; 2 Department of Biochemistry, Microbiology and Immunology, University of Tennessee Health Science Center, Memphis, Tennessee, United States of America; 3 Department of Surgery at the University of Tennessee Health Science Center, Memphis, Tennessee, United States of America; 4 Joint Program of Biomedical Engineering at the University of Tennessee Health Science Center and the University of Memphis, Memphis, Tennessee, United States of America; 5 CirQuest Labs, LLC, Memphis, Tennessee, United States of America; Vanderbilt University Medical Center, United States of America

## Abstract

Tumor cell metastasis, a process which increases the morbidity and mortality of cancer patients, is highly dependent upon matrix metalloproteinase (MMP) production. Small molecule inhibitors of MMPs have proven unsuccessful at reducing tumor cell invasion *in vivo*. Therefore, finding an alternative approach to regulate MMP is an important endeavor. Tetraspanins, a family of cell surface organizers, play a major role in cell signaling events and have been implicated in regulating metastasis in numerous cancer cell lines. We stably expressed tetraspanin CD9 in an invasive and metastatic human fibrosarcoma cell line (CD9-HT1080) to investigate its role in regulating tumor cell invasiveness. CD9-HT1080 cells displayed a highly invasive phenotype as demonstrated by matrigel invasion assays. Statistically significant increases in MMP-9 production and activity were attributed to CD9 expression and were not due to any changes in other key tetraspanin complex members or MMP regulators. Increased invasion of CD9-HT1080 cells was reversed upon silencing of MMP-9 using a MMP-9 specific siRNA. Furthermore, we determined that the second extracellular loop of CD9 was responsible for the upregulation of MMP-9 production and subsequent cell invasion. We demonstrated for the first time that tetraspanin CD9 controls HT1080 cell invasion via upregulation of an integral member of the MMP family, MMP-9. Collectively, our studies provide mounting evidence that altered expression of CD9 may be a novel approach to regulate tumor cell progression.

## Introduction

Tumor cell invasion into the surrounding extracellular matrix (ECM) increases the morbidity and mortality of all forms of cancer. ECM degradation is a prerequisite for tumor cell invasion and is mediated by matrix metalloproteinase (MMP) production and activity. MMPs are a family of zinc-dependent endopeptidases that degrade specific components of the ECM (reviewed in [1], [2]). Targeting tumor cell invasion is paramount to suppressing tumor cell motility and eventual metastasis. Unfortunately, clinical trials using small-molecule inhibitors to block MMP activity have not been successful [3]–[6]. Therefore, other means to control MMP production and activity must be identified in order to regulate invasive cell phenotypes.

An interesting link between tetraspanins and cancer metastasis has been demonstrated in multiple cell lines [7], [8].Tetraspanins are four-membrane spanning proteins that most notably function as cell surface protein organizers through tetraspanin-enriched membrane complexes [9]. Determining the role of tetraspanins in either promoting or suppressing tumor metastasis is complicated by the number and function of molecules that complex with tetraspanins, including other tetraspanins and integrins [10], [11]. One member of the tetraspanin family, CD9, is particularly important in tumor cell biology [12]. There is an increasing amount of evidence that supports a role for CD9 in metastatic cancers both *in vitro* and *in vivo* [13]–[15]. *In vivo* studies suggested that increased CD9 expression correlated with advanced gastric cancer and a poor prognosis [16], [17]. Another study conducted on prostate carcinoma progression noted a significant increase in CD9 expression in primary, metastasizing adenocarcinoma compared to advanced, non-metastasizing adenocarcinomas [18]. Strong CD9 expression in metastasizing cancers indicates that this protein may be a novel target for regulating the invasive phenotype of these cells.

Tetraspanins may regulate the invasive process of cancer cells by controlling the expression, release, and activity of MMP and tissue inhibitors of metalloproteinases (TIMPs). Data imply that CD63 [19] and CD151 [20] regulate MT1-MMP activity either by proteolysis or association, respectively. CD63 also interacts with TIMP-1 at the cell surface to regulate its activity in human breast epithelial cells [21]. Furthermore, double deficiency of both CD9 and CD81 resulted in increased amounts of MMP-2 and MMP-9 in a macrophage cell line [22], and CD151 played a role in activating pro-MMP-7 in osteoarthritic chondrocytes [23]. It is well established that CD9 overexpression decreases cell motility in most cancerous cell lines [24]–[26]; however, there is notable ambiguity on the effect CD9 may have on the invasive cell phenotype by regulating MMP and TIMP production.

We studied exogenous CD9 expression in human fibrosarcoma (HT1080) cells, a widely used *in vitro* metastasis model for cell invasion [27]–[30]. This stably transfected cell line was used to address the consequences of CD9 expression on the expression of other tetraspanin-enriched complex members and on the invasive capabilities of these cells. Significant findings from our study demonstrate that CD9-HT1080 cells displayed a highly invasive phenotype compared to their Mock transfected counterparts. CD9 expression was directly correlated with MMP-9 expression, and the suppression of MMP-9 alone was sufficient to negate the increased invasive phenotype of CD9-HT1080 cells. Furthermore, the second extracellular loop of CD9 was critical for the observed increase in MMP-9 and cell invasion. Our study confirms that the tetraspanin CD9 serves to regulate HT1080 cell invasion via upregulation of MMP-9.

## Materials and Methods

### Reagents and Antibodies

Dulbecco’s modified Eagle’s medium (DMEM), fetal bovine serum (FBS), penicillin-streptomycin, trypsin-EDTA, Geneticin (G418), and human plasma fibronectin (FN) were purchased from Gibco (Grand Island, NY). A murine monoclonal antibody specific for the second extracellular loop of CD9 (mAb7) was previously generated in our laboratory [31]. A rabbit polyclonal antibody specific for the first extracellular loop of CD9 (Rap2) was also generated in our laboratory and previously reported [32].Anti-CD63 and anti-CD151 antibodies were purchased from BD Pharmingen (San Diego, CA). Anti-CD81, anti-α2, anti-α4, anti-α5, anti-α6, and anti-β1 (TS2/16) antibodies were from Santa Cruz Biotechnology (Santa Cruz, CA). Matrigel from Engelbreth-Holm-Swarm mouse tumor and 8.0 µm pore cell culture inserts were purchased from BD Biosciences (Bedford, MA). Lipofectamine 2000 transfection reagent was purchased from Invitrogen (Carlsbad, CA). All other reagents were purchased from Sigma Aldrich (St. Louis, MO).

### Cell Culture and Transfection

Human fibrosarcoma (HT1080) cells were purchased from American Type Culture Collection (Manassas, VA) and cultured in DMEM supplemented with 10% FBS and 1% penicillin-streptomycin solution. Wild type HT1080 cells were transfected by electroporation with either the control pRC/CMV plasmid (Mock), the pRC/CMV plasmid containing full-length human CD9 cDNA insert (CD9), or the pRC/CMV plasmid containing CD9 without the second extracellular loop amino acids 173–192 (Δ6, described in [32]). To obtain stable transfectants, transfected cell populations were selected by the addition of media containing Geneticin (G418, 0.75 mg/ml). All cells were cultured in a humidified, 5% CO_2_, 37°C incubator.

### RNA Isolation and qRT-PCR Analysis

Forward and reverse primers were designed using Universal Probe Library primer design tool and were purchased from Sigma Aldrich (Table S1, S2). Primer efficiencies were tested on universal human RNA, and were only used if the efficiency was greater than 1.80. Total cellular RNA was isolated from Mock- and CD9-HT1080 cells using the RNeasy isolation kit (Qiagen, Valencia, CA) according to the manufacturer’s instructions. The quality of the RNA was assessed using an Agilent Bioanalyzer 2100 (Santa Clara, CA). All samples had an RNA integrity number of 10. RNA quantity in the isolated samples was estimated using a nanodrop spectrophotometer (Thermo Scientific, Rockford, IL), and 1 µg of total RNA was subjected to reverse transcription using the transcriptor first-strand cDNA synthesis kit (Roche, Indianapolis, IN). The resulting cDNA was subsequently used for analysis by qRT-PCR using TaqMan chemistry (Roche) and a Lightcycler 480 system at the Molecular Resource Center (University of Tennessee Health Science Center, Memphis, TN). Sample tests were run in triplicate, and the resulting average cycle threshold (CT) values were normalized to cyclophilin-D housekeeping gene (ΔCT). The ΔCT values for Mock HT1080 cells were subtracted from CD9-HT1080 values (ΔΔCT). Fold changes in CD9-HT1080 mRNA relative to Mock HT1080 mRNA were calculated by 2^−ΔΔCT^. Fold changes greater than 2 or less than 0.5 were considered significant.

### Flow Cytometry

Mock- and CD9-HT1080 cells were harvested and suspended at 5.0×10^5^ cells/ml in 5% goat serum-DMEM (blocking media) and incubated on ice for 45 minutes. All subsequent antibody incubations were performed on ice. Primary antibody was added (4 µg/ml) and incubated for 1 hour. Unbound primary antibody was removed by washing the cells three times. Briefly, the cells were pelleted (800 g for 5 minutes), the supernatant was discarded, and the cell pellet was resuspended using 1 ml of ice-cold PBS (pH 7.4). The cells were incubated with secondary FITC-conjugated antibody (5 µg/ml) in blocking media for 1 hour. The secondary antibody was removed by washing, and the cells were suspended in PBS for data acquisition. Analysis of the data was performed using a FACS Calibur flow cytometer equipped with Cell Quest Pro software (Beckton-Dickinson, Bedford, MA). The geometric mean fluorescence intensity values were averaged among three or more independent experiments.

### Matrigel Invasion Assay

Matrigel diluted to 250 µg/ml was added inside cell culture inserts and allowed to solidify at 37°C for 1 hour. 1×10^5^ Mock- and CD9-HT1080 cells were added to separate cell culture inserts, and 1% FBS-DMEM was added to the bottom well as a chemoattractant. As a control, equal amounts of cells were added to uncoated cell culture inserts. The 24-well tissue culture plates were placed in a humidified, 5% CO_2_, 37°C incubator. After 20 hours incubation, the inside of the tissue culture insert was cleared of cells by scrubbing with a cotton swab. The cells on the bottom of the insert were fixed in ice-cold 100% methanol for 2 minutes, rinsed twice with PBS, and stained using 0.05% crystal violet for 30 minutes. After rinsing with PBS, the culture insert membranes were cut out and mounted onto microscope slides with Permount. Cells were counted in 10 random fields of view per membrane. Cell counts in each field of view were averaged. Percent cell invasion was calculated by dividing the number of cells that invaded through matrigel-coated inserts by the number of cells that migrated through uncoated inserts and multiplied by 100. Relative invasion was calculated by normalizing the percent cell invasion to the corresponding control.

### Gelatin zymography

For zymography analysis, equal numbers of transfected-HT1080 cells were seeded onto 10 µg/ml of FN-coated cell culture plates in serum-free DMEM. Cells were allowed to adhere for 24 to 48 hours. Culture supernatants were harvested and clarified by a two-step centrifugation at 4°C (first at 800 g and second at 14,000 g). The resulting culture supernatant was mixed with non-reducing sample buffer and subjected to electrophoresis on 10% SDS-polyacrylamide gels containing 1 mg/ml of porcine gelatin (Sigma Aldrich). After electrophoresis, SDS was removed by washing gels with 2.5% (v/v) Triton X-100 solution. The gels were incubated in digestion buffer (50 mM Tris, 200 mM NaCl, and 10 mM CaCl_2_) for 14 hours at 37°C and then fixed in a solution containing 50% ethanol and 10% acetic acid for 2 hours at room temperature. The gels were then washed twice in 50% methanol and 10% acetic acid solution and stained with 0.1% Coomassie brilliant blue R-250 in 20% methanol and 10% acetic acid solution for 2 hours. Lastly, the gels were destained with 50% methanol and 10% acetic acid solution, scanned, and subjected to densitometry analysis using NIH Image J software. Relative density was calculated by dividing the intensity of the MMP-9 band by the MMP-2 band, then normalizing the data to the corresponding Mock-HT1080 bands.

### ELISA

ELISA kits for MMP-1 and MMP-9 were purchased from RayBiotech, Inc (Norcross, GA) and eBioscience (San Diego, CA), respectively. Analysis of pro- and active-proteinase expression was performed per the manufacturer’s protocol using cleared supernatant from Mock- and CD9-HT1080 cells incubated for 24 to 48 hours on 10 µg/ml FN-coated cell culture plates in serum-free DMEM. Samples were run in duplicate, and results were compared to a curve generated using reconstituted protein standards supplied with each kit. The lower limits of detection for MMP-1 and MMP-9 were less than 8 pg/ml and 50 pg/ml, respectively. The calculated overall intra-assay coefficients of variation were less than 10%.

### Knockdown of MMP-9

Small interfering RNA (siRNA) oligomer duplexes targeting MMP-9 were ordered from Sigma Aldrich (St. Louis, MO). Three different MMP-9 siRNA were initially screened for their effects on tetraspanin, integrin, and MMP expression. One siRNA was successful at silencing MMP-9 expression without affecting other tetraspanins, integrins, and MMPs pertinent to our study and was chosen for subsequent experiments. A non-specific, scrambled siRNA was used as a control. Transfection was performed on a subconfluent monolayer of Mock- or CD9-HT1080 cells using Lipofectamine 2000 transfection reagent (Invitrogen). After 6 hours incubation with siRNA and Lipofectamine 2000, the cell monolayer was washed and the cells were incubated with complete media. The cells were harvested and used in subsequent experiments 48 hours after transfection.

### Statistical Analysis

Experiments were repeated at least three independent times. Statistical analysis was carried out using SPSS software. The values are expressed as mean ± standard deviation. An independent samples t-test or a Mann-Whitney U Test was used to compare two means. Analysis of Variance (ANOVA) was used to compare three means of normally distributed homogenous data. A Tukey’s HSD post-hoc analysis was conducted to determine any difference among the groups. A Welch’s ANOVA was used for three sample means with non-normally distributed data, and a Dunnet’s T3 post-hoc analysis was used to determine any difference among these means. Graphs were generated using GraphPad Prism 6 software. A *p* value less than 0.05 was considered statistically significant.

## Results

### CD9 Overexpression in HT1080 Cells did not Alter the Expression of other Tetraspanin Family Members

**Figure 1:**
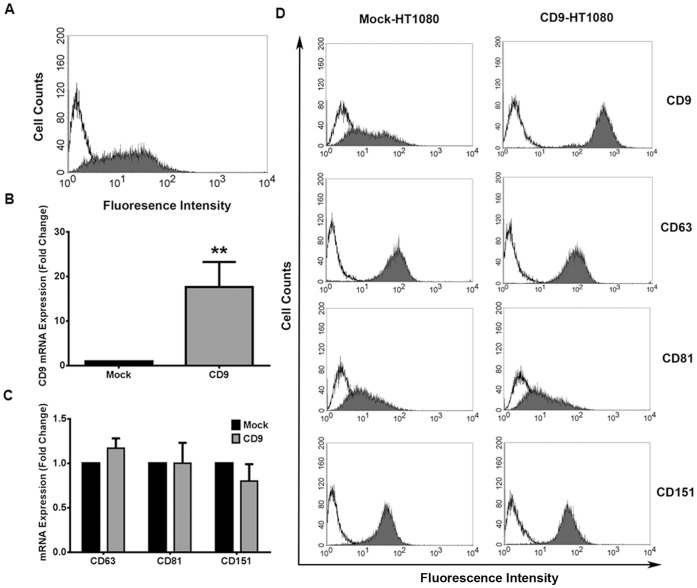
Overexpression of CD9 in HT1080 cells does not alter the expression of other tetraspanins. (**A**) The cell surface expression of CD9 on wild-type HT1080 cells was assessed using flow cytometric analysis after binding a monoclonal antibody specific for CD9 (mAb7, shaded histogram). A non-specific isotype-matched antibody (IgG) was used as a negative control (unshaded histogram). (**B, C**) Total RNA was collected from Mock- and CD9-HT1080 cells, reverse transcribed to cDNA, and probed using primers specific for integral tetraspanins using qRT-PCR. The fold change in mRNA expression of tetraspanins was calculated from resulting cycle threshold values normalized to cyclophilin D. (**D**) Flow cytometry was used to assess the cell surface expression of key tetraspanins on the cell surface of Mock- (left panels) and CD9- (right panels) HT1080 cells **, p<0.001.

Human fibrosarcoma (HT1080) cells endogenously expressed low levels of CD9 on their cell surface as indicated by flow cytometry (Fig. 1A). A population of CD9-expressing HT1080 cells was generated by transfecting wild-type HT1080 cells with a plasmid containing a full-length CD9 insert. Likewise, the same plasmid without the CD9 insert was used to generate Mock-HT1080 cells. Stably transfected cell colonies were generated by multiple passages of these transfected cells in Geneticin-containing selection media. CD9 mRNA levels were upregulated approximately 20-fold in CD9-HT1080 compared to Mock-HT1080 cells (Fig. 1B). The mRNA levels of other constitutively expressed members of the tetraspanin family – including CD63, CD81, and CD151– were not significantly changed as a result of CD9 expression (Fig. 1C).

Flow cytometry was further used to confirm that changes in mRNA expression corresponded with the proper trafficking and expression of tetraspanins on the cell surface. Also, increased CD9 expression may alter the cell surface expression of other tetraspanins due to rearrangement of the tetraspanin-enriched microdomain. Therefore, we observed changes in the cell surface expression of tetraspanins and found a prominent increase in the expression of CD9 at the cell surface of CD9-HT1080 cells relative to the Mock transfected HT1080 cells (Fig. 1D). The mean fluorescence intensity was 11.4±2.3 and 538.8±149.1 for Mock- and CD9-HT1080 cells, respectively (p<0.001). Exogenous expression of CD9 did not significantly affect the cell surface expression of CD63, CD81, or CD151 (Fig. 1D). Thus, any phenotypic differences between Mock- and CD9-HT1080 cells may be attributed directly to upregulation of CD9 expression.

**Figure 2:**
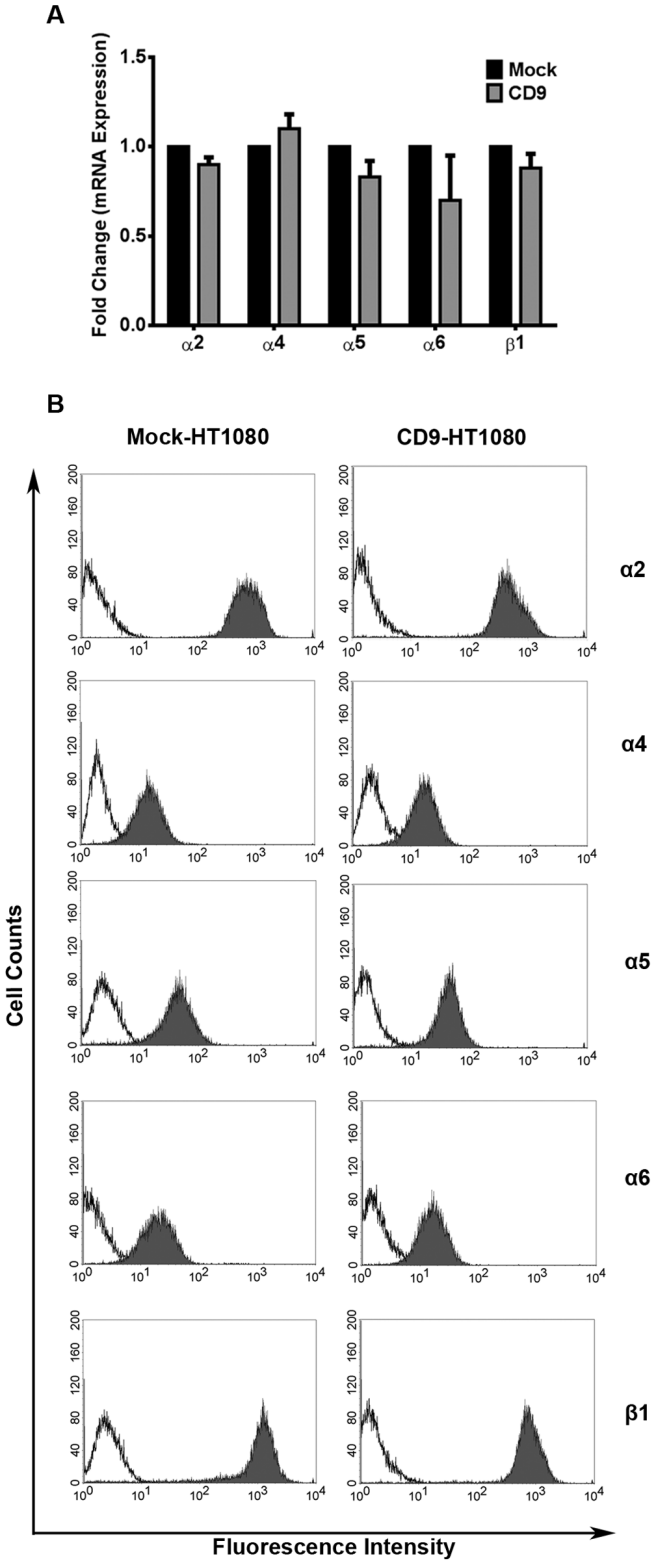
CD9-HT1080 cells have stable mRNA expression of integrins though α2 and β1 are reduced at the cell surface. (**A**) The fold change in mRNA expression of integrins was compared between Mock- and CD9-HT1080 cells using qRT-PCR. (**B**) Cell surface expression of the same integrin subunits (shaded histograms) was evaluated by flow cytometry. A non-specific isotype-matched antibody (IgG) was used as a negative control (unshaded histograms).

Other major regulators of activity within tetraspanin-enriched microdomains include integrins. We explored whether or not the mRNA expression of integrins α2, α4, α5, α6, and β1 were significantly different between Mock- and CD9-HT1080 cells. CD9 expression did not result in any fluctuations in the mRNA production of integrins α2, α4, α5, α6, or β1 (Fig. 2A). Furthermore, we determined that the cell surface expression of integrin subunits α4, α5, and α6 was unaffected by CD9 overexpression (Fig. 2B). However, the cell surface expression of α2 and β1 integrin subunits was lower in CD9 transfectants (Fig. 2B). CD9-HT1080 α2 fluorescence intensity values were 1.4 fold less and β1 values were 1.2 fold less than Mock-HT1080 values (p<0.001, and p = 0.002, respectively). These findings suggest a small though statistically significant decrease in the integrin heterodimer α2β1 at the cell surface.

### CD9-HT1080 Cells have Increased Cell Invasion and MMP-9 Production and Release

**Figure 3:**
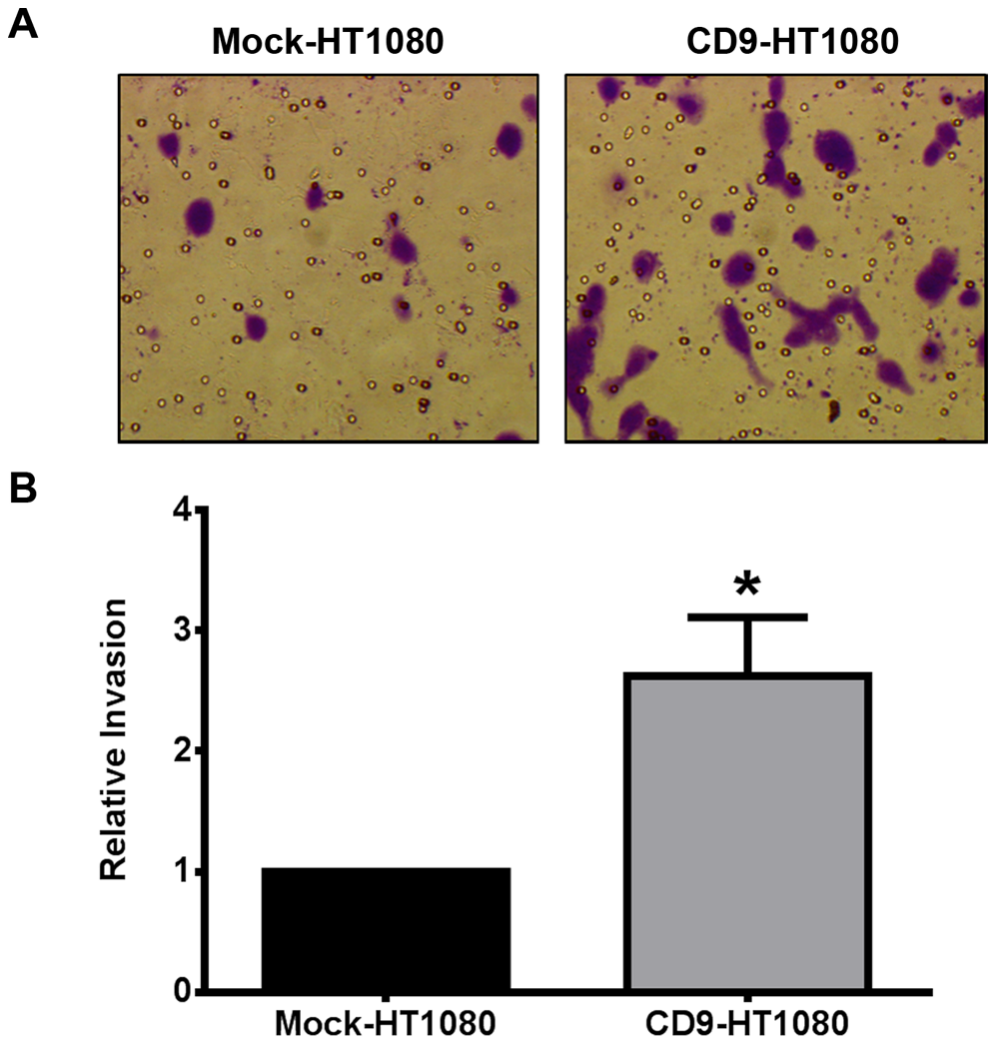
The invasive phenotype of HT1080 cells is increased upon CD9 overexpression. A matrigel invasion assay was used to assess the invasive properties of Mock- and CD9-HT1080 cells. Cells were allowed to invade through matrigel and adhere to the bottom of a cell culture insert as detailed in the Materials and methods section. (**A**) A representative image of stained cells after 20 hours of invasion though matrigel. (**B**) Invasion through matrigel-coated inserts was compared to cells that invaded through uncoated inserts and results shown are normalized to Mock-HT1080 cell invasion *, p<0.05.

Tetraspanins are known to regulate the metastatic phenotype of tumor cells 7,8. A matrigel invasion assay was used to establish any changes in the invasive phenotype of CD9-HT1080 cells. After 20 hours of invasion, we observed 45% more CD9-HT1080 cells migrated through matrigel-coated cell culture inserts compared to Mock-HT1080 cells (p = 0.002; Fig. 3A, 3B). This outcome indicates that the presence of CD9 on the cell surface contributes to an increase in the invasive capabilities of HT1080 cells.

**Figure 4:**
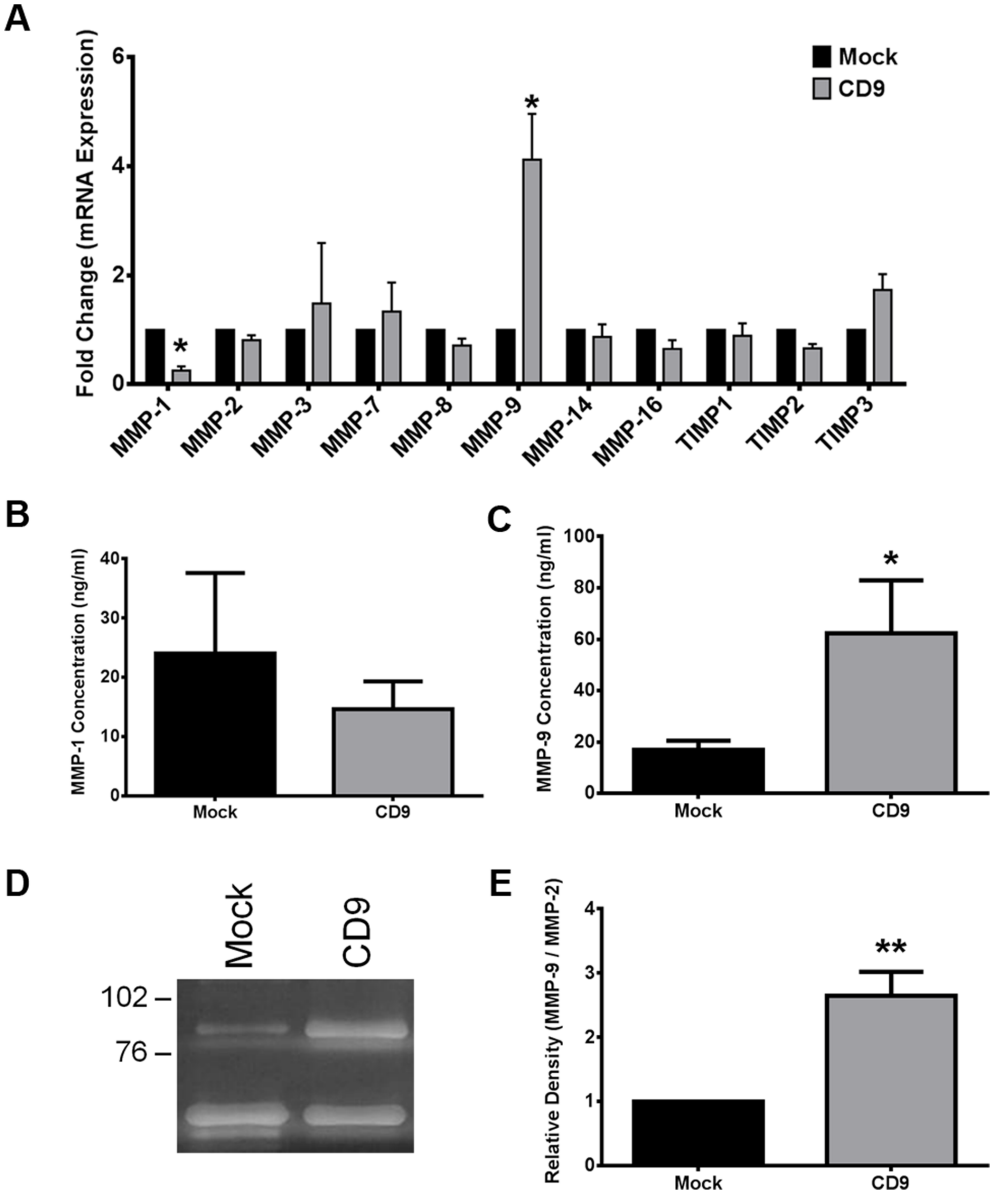
MMP-9 expression and release are greatly enhanced in CD9-HT1080 cells. (**A**) Fold changes in MMP and TIMP mRNA expression for Mock- and CD9-HT1080 cells were calculated from cycle threshold values using qRT-PCR. (**B, C**) Specific ELISA kits were used to measure the concentration (ng/ml) of pro- and active- MMP-1 and MMP-9 in the cleared supernatant of Mock- and CD9-HT1080 cells. (**D**) A representative gelatin zymogram of Mock- and CD9- HT1080 cell conditioned media. The absence of Coomassie staining at 92 kDa indicates the presence of pro-MMP-9 and at 72 kDa represents pro-MMP-2. (**E**) Quantification of the relative band intensity was calculated by densitometry analysis as described in the Materials and methods section *, p<0.05; **, p<0.001.

Invasion of cancer cells is tightly regulated by a balance in MMP and TIMP production [4]. Tetraspanins have previously been demonstrated to regulate MMP and TIMP expression in other cell lines [22], [23], [33], [34]. We used qRT-PCR to examine if CD9 overexpression had any effect on relative MMP or TIMP mRNA levels. Our results indicated a 4.1-fold increase in the production of MMP-9 mRNA in cells overexpressing CD9 compared to Mock (Fig. 4A). We also observed a 3.9-fold decrease in the expression of MMP-1 mRNA. The mRNA expression levels of other MMPs and TIMPs including MMP-2, MMP-3, MMP-7, MMP-8, MMP-14 (MT1-MMP), MMP-16 (MT2-MMP), TIMP-1, TIMP-2, and TIMP-3 were unchanged between Mock- and CD9-HT1080 cells (Fig. 4A). Upon using multiple different and efficient primers, TIMP-4 mRNA expression was not detected in either Mock- or CD9-HT1080 cells.

MMP-1 and MMP-9 specific ELISAs were used to establish if the observed mRNA changes resulted in changes in soluble proteinase release into the media. MMP-1 proteinase concentration in the supernatants from CD9-HT1080 cells tended to be less than that detected in the Mock cells; however, this change was not statistically significant (Fig. 4B). Consistent with increased MMP-9 mRNA levels, MMP-9 proteinase concentration in the supernatant of CD9-HT1080 cells was 57% higher than Mock-HT1080 cells (p = 0.016; Fig. 4C). There was a very strong direct correlation between the levels of CD9 mRNA expression and the proteinase concentration of MMP-9 in the CD9-HT1080 cell supernatant after 48 hours (Pearson correlation coefficient = +0.973, p = 0.027). These findings suggest the involvement of CD9 in the induction of MMP-9 mRNA synthesis and release into the supernatant.

We confirmed any changes in the secretion of gelatinase MMP-9 in cell culture supernatants using a gelatin zymogram. Pro-MMP-9 gelatinolytic band intensity was augmented in the cell culture supernatant of CD9-HT1080 cells (Fig. 4D). The 72 kDa pro-MMP-2 gelatinolytic band that served as an internal control was similar in both Mock- and CD9-HT080 conditioned media. Quantification of the relative density of the enzymatic bands established that pro-MMP-9 secretion was two-fold greater in CD9-HT1080 cells compared to Mock cells (p<0.001; Fig. 4E). The amount of pro-MMP-2 in the cell supernatant was not significantly different between Mock- and CD9-HT1080 cells.

### Increased CD9-HT1080 Cell Invasion is a Consequence of Increased MMP-9 Production

**Figure 5:**
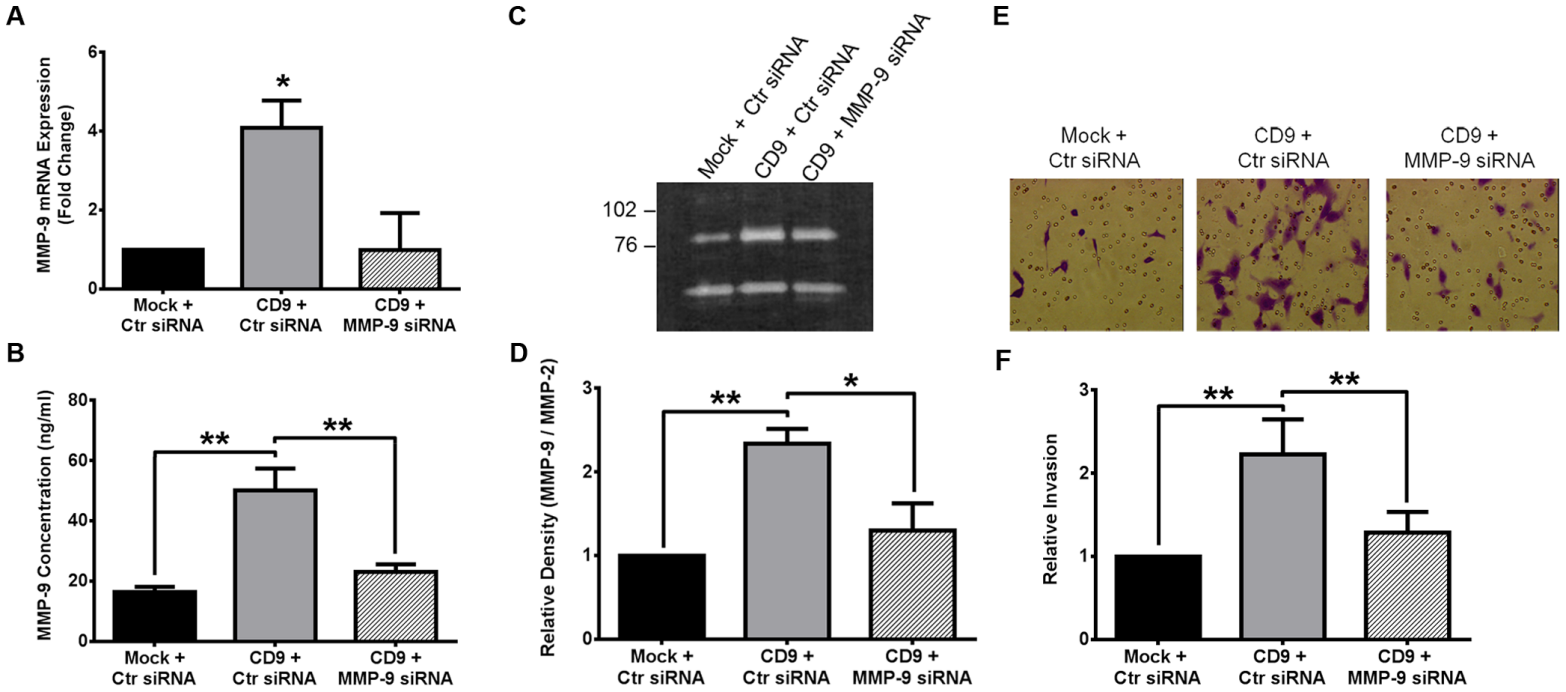
Silencing MMP-9 in CD9-HT1080 cells is sufficient to suppress the invasive phenotype. CD9-HT1080 cells were transiently transfected with short-interfering RNA directed to MMP-9 (CD9+ MMP-9 siRNA). Likewise, Mock- and CD9- HT1080 cells were transfected with a scrambled siRNA as a negative control (Mock+Ctr siRNA or CD9+ Ctr siRNA). (**A**) qRT-PCR analysis was used to measure changes in MMP-9 mRNA levels among Ctr and MMP-9 siRNA transfected cells. (**B**) Changes in release of MMP-9 (ng/ml) in to the cleared culture supernatant were examined using a MMP-9 specific ELISA kit. (**C**) Pro-MMP-9 levels in the supernatants of transiently transfected cells was measured using gelatin zymography and (**D**) quantified by densitometry. (**E**) A representative image of cells invading though matrigel after transfection with siRNA. (**F**) Percent cell invasion through matrigel-coated inserts after 20 hours was quantified and the results were normalized to Mock+Ctr siRNA treated cells *, p<0.05; **, p<0.001.

We hypothesized that increased MMP-9 mRNA expression and proteinase release into the media was responsible for the increased invasive cell phenotype observed in the CD9-HT1080 cells. To test this hypothesis, we utilized a short-interfering RNA-mediated silencing approach to transiently knock down MMP-9 expression in CD9-HT1080 (CD9+MMP9 siRNA). A scrambled control siRNA was transfected into Mock- and CD9-HT1080 cells and used as a control (Mock+Ctr siRNA and CD9+Ctr siRNA). Upon transfection, there was significantly less MMP-9 mRNA expression in CD9+MMP-9 siRNA compared to CD9+Ctr siRNA (p = 0.005; Fig. 5A). The mRNA expression levels of CD9, CD81, CD151, MMP-1, and MMP-2 were unchanged upon transfection with MMP-9 siRNA (data not shown). Treatment with MMP-9 siRNA in CD9-HT1080 cells resulted in 46% decrease MMP-9 proteinase concentration in the conditioned media compared to CD9+Ctr siRNA as measured by ELISA (p<0.001; Fig. 5B). There was no significant difference in the MMP-9 concentration of Mock+Ctr siRNA and CD9+MMP9 siRNA HT1080 cell conditioned media. These results confirm that silencing of MMP-9 in CD9-HT1080 cells lowers the expression and release of MMP-9 to that observed in Mock-HT1080 cells.

Gelatin zymography analysis of cell supernatants fully supported our qRT-PCR and ELISA findings. There was a significant difference in the amount of pro-MMP-9 in the cell culture supernatant between the three groups of transiently transfected cells. CD9+MMP-9 siRNA gelatin degradation decreased 51% compared to CD9+Ctr siRNA (p<0.001; Fig. 5C, 5D). However, there was no significant difference in gelatinolytic band intensity between Mock+Ctr siRNA and CD9+MMP-9 siRNA HT1080 cells (Fig. 5D). Moreover, an invasion assay was used to determine the effect of silencing MMP-9 in CD9-HT1080 cells. There was a 58% reduction in the invasive capabilities of CD9+MMP-9 siRNA HT1080 cells compared to CD9+Ctr cells (Fig. 5E, 5F). No statistical difference was observed between the relative invasion of Mock+Ctr and CD9+MMP-9 siRNA HT1080 cells. These results support the concept that the expression of MMP-9 elicited by overexpression of CD9 is responsible for the observed invasive phenotype of CD9-HT1080 cells.

### CD9 Second Extracellular Loop Contributes to Increases in MMP-9 Expression

**Figure 6:**
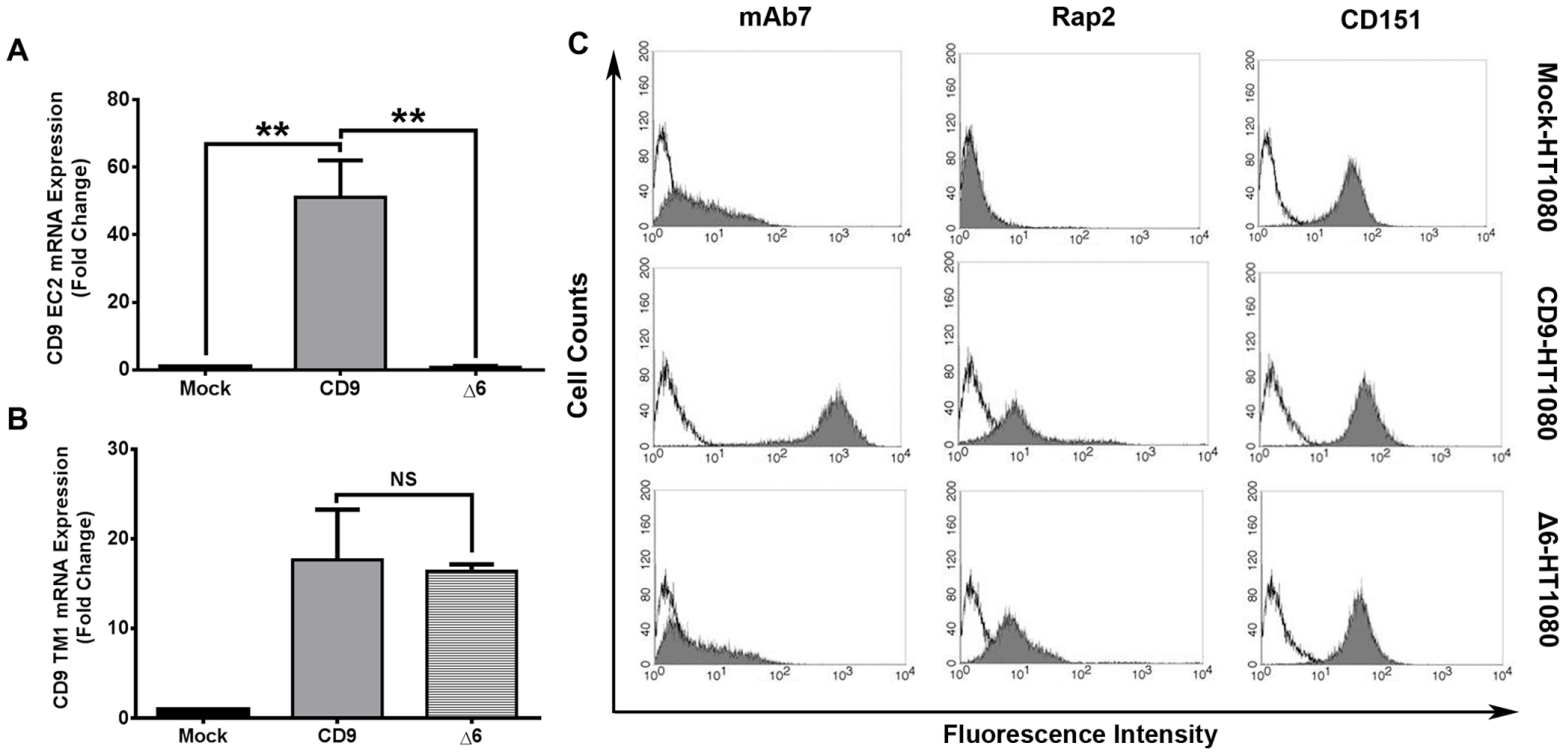
Characterization of the second extracellular loop (EC2) deletion mutant of CD9 in HT1080 cells. Upon transfection with the EC2 deletion mutant in HT1080 cells, mRNA expression was measured using primer pairs specific for the nucleotides coding the EC2 (**A**) and TM1 (**B**) regions of CD9. (**C**) Flow cytometric analysis of Mock-, CD9-, and Δ6-HT1080 cells is indicated by each row, and each column indicates CD9 EC2 (mAb7), CD9 TM1 (Rap2), or CD151 binding (shaded histograms) **, p<0.001.

Previously, our laboratory and others have attributed major functions of CD9 to the second extracellular loop (EC2) of the protein 32,35–37. Expanding the scope of our study, we hypothesized that increases in MMP-9 expression and release into the media may be attributed to the highly functional EC2 of CD9. We stably transfected wild-type HT1080 cells with a CD9 mutant lacking EC2 amino acids 173–192 (Δ6-HT1080). Primers were designed to detect the levels of mRNAs that code for the amino acids at the N-terminal and first transmembrane region (TM1; amino acids 5 to 35) and the second extracellular loop of CD9 (EC2; amino acids 158–185). Upon transfection, we were unable to detect CD9 EC2 mRNA in Δ6-HT1080 cells; however, the levels of CD9 EC2 mRNA were not significantly different between Mock- and Δ6-HT1080 cells (Fig. 6A). CD9 TM1 mRNA levels were not significantly different between CD9- and Δ6-HT1080 cells (Fig. 6B). We confirmed that the EC2 deletion mutant was expressed on the cell surface using flow cytometry. As seen in the left column of Figure 6C, the binding of mAb7 – a monoclonal antibody specific for CD9 EC2 – was similar for both Mock- and Δ6-HT1080 cells. Binding of the anti-CD9 EC1 antibody, Rap2, was not detectable in Mock-HT1080 cells, but was similar in CD9- and Δ6-HT1080 cells (Fig. 6C, middle column). Anti-CD151 antibody bound similarly to all three cell lines.

**Figure 7:**
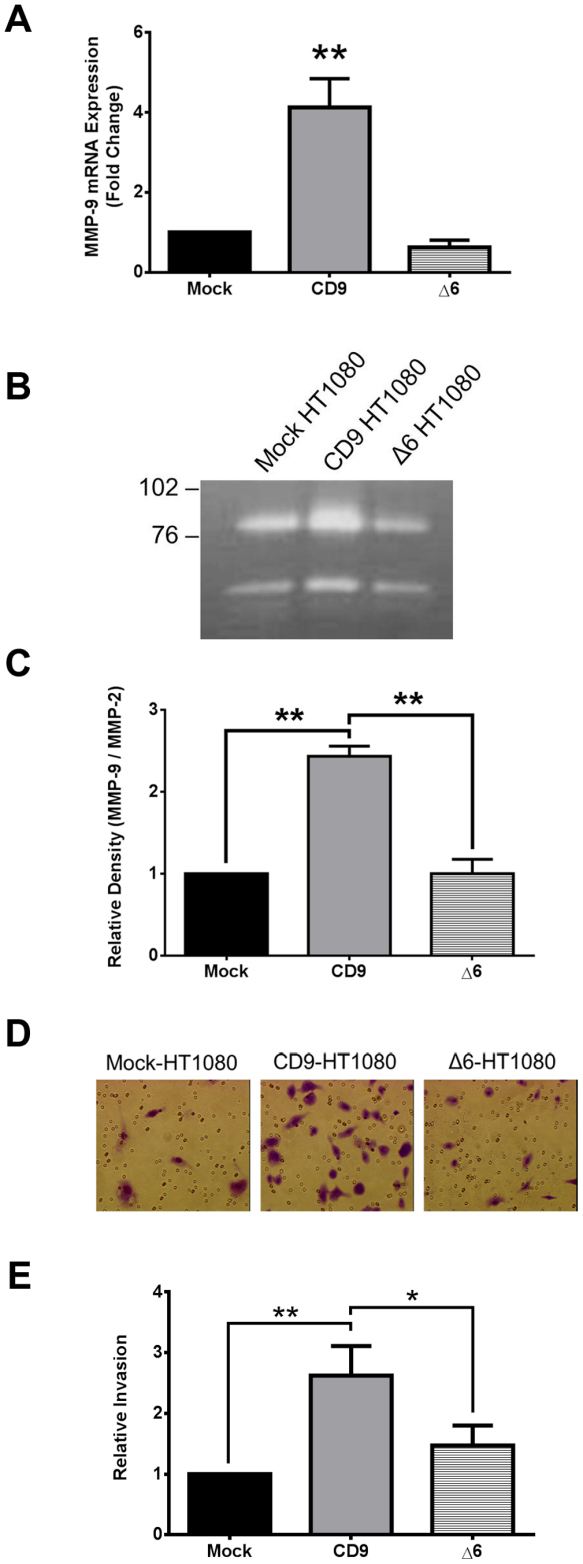
An increase in MMP-9 expression and release and subsequent cell invasion requires the second extracellular loop (EC2) of CD9. (**A**) MMP-9 mRNA expression was measured in Mock-, CD9-, and Δ6-HT1080 cells using qRT-PCR (**B,C**) Release of pro-MMP-9 and pro-MMP-2 was measured by gelatin zymography and quantified using Image J. (**D,E**) A representative picture of cell invasion through matrigel coated inserts and relative quantification of cell invasion *, p<0.05; **, p<0.001.

We found MMP-9 mRNA levels to be 7.8 fold lower in Δ6-HT1080 compared to CD9-HT1080 cells (p<0.001; Fig. 7A). MMP-2 mRNA levels between Δ6-HT1080 and CD9-HT1080 cells were unchanged. The concentration of MMP-9 in the supernatant of Δ6-HT1080 cells (11.9±1.6 ng/ml) was not significantly different from Mock-HT1080 revealed earlier (16.9±3.5 ng/ml). Quantification of MMP-9 gelatin degradation of Δ6-HT1080 cells was 33% lower than degradation observed in CD9-HT1080 cells (Fig. 7B). However, there was no significant difference in MMP-9 gelatinolytic band intensity between Mock- and Δ6-HT1080 cells (Fig. 7C). MMP-2 degradation was similar among Mock-, CD9- and Δ6-HT1080 cells. A matrigel invasion assay using these cell lines revealed that Δ6-HT1080 cells were significantly less invasive than CD9-HT1080 cells (Fig. 7D, 7E). There was no significant difference between the invasive properties of Mock- and Δ6-HT1080 cells. These data suggest a specific role for the second extracellular loop of CD9 in the induction of MMP-9 expression, release, and degradation.

## Discussion

We utilized an invasive and metastatic human carcinoma cell line to explore the contribution of tetraspanin CD9 to tumor cell invasion. We stably expressed CD9 in HT1080 cells at mRNA and cell surface levels similarly to the endogenous expression of CD9 in other cancerous cell lines including colon [38] and non-small cell lung cancer [25]. Tetraspanins function at the cell surface through organizing multimolecular complexes composed of tetraspanins and associating molecules, including integrins [9], [10]. Upon expression of a single tetraspanin, the expression levels of other tetraspanins and integrins may fluctuate at the mRNA or cell surface level. This rarely explored phenomenon may explain why CD9 function in various cancer cell lines is not fully congruent [14], [39], [40]. Our studies indicated increased CD9 at the cell surface level was not sufficient to stimulate or suppress mRNA expression of other integral members within the functional tetraspanin microdomain. The cell surface expression of other tetraspanin members that play prominent roles in cancer cell invasion including CD63 [41], CD81 [22], [33], [42], and CD151 [43]–[45] were unaffected by CD9 overexpression. These findings suggest the increased invasive phenotype of CD9-HT1080 cells may be attributed primarily to increases in CD9.

Interestingly, there was a significant decrease in the cell surface levels of both α2 and β1 integrins in CD9-HT1080 cells compared to Mock cells. CD9 expression may downregulate the cell surface expression of the α2β1 integrin subunit. There is convincing evidence that MMP-1 expression is induced only in osteogenic cell lines that express α2β1 [46]. Moreover, blockade with either MMP-1 or α2 antibodies significantly decreased keratinocyte migration, indicating that α2 may regulate MMP-1 expression [47]. We conclude that decreased MMP-1 mRNA expression observed in CD9-HT1080 cells may be related to reduced α2β1 cell surface expression. However, our findings at the mRNA level did not fully translate to a significant decrease in MMP-1 proteinase release in the cell supernatant (Fig. 4B). Therefore, we did not pursue α2β1 or MMP-1 as a mediator of phenotypic differences between Mock- and CD9-HT1080 cells.

We concluded that the invasive phenotype observed in CD9-HT1080 cells was the result of significant increases in MMP-9 mRNA expression and proteinase release into the media. This conclusion was confirmed by silencing MMP-9 in CD9-HT1080 cells, which consequently reverted the invasive phenotype of these cells to mimic that of the Mock-transfected cells. An earlier study did not observe this invasive phenotype in CD9-HT1080 cells using a matrigel invasion assay [48]. This difference may be the result of higher cell numbers and increased chemoattractant the earlier study used in the invasion assays, which would have overwhelmed the possibility of observing any differences in cell invasion. However, when using conditions more similar to our experimental design, Zvieriev et al. demonstrated that exogenous CD9 expression increased *in vitro* matrigel invasion in a prostate cancer cell line [49]. A recent study corroborated our findings that silencing of MMP-9 in HT1080 cells decreases their invasive capabilities [50].

Other laboratories have demonstrated that tetraspanin expression may indirectly result in the regulation of MMP production by influencing TIMP expression and activity. Jee and colleagues demonstrated that KAI1/CD82 upregulated TIMP-1 to suppress tumor cell invasion in lung carcinoma model [51]. TIMP-1 complexes with pro-MMP-9 and inhibits its conversion to the active proteinase [52]. Furthermore, tetraspanin CD63 interacts with TIMP-1 at the cell surface to regulate cell survival and apoptosis in a breast epithelial cell line [21]. Interestingly, TIMP-4 expression was not detectable in either Mock- or CD9-HT1080 cells. This finding is most likely due to the fact that TIMP-4 expression is prominent only in the brain, heart, ovary, and skeletal muscles of adult mice [53] and was originally isolated in the human heart with very low or absent levels of expression in other major organs [54]. The absence of a significant change in the mRNA expression of TIMP-1, TIMP-2, or TIMP-3 upon CD9 overexpression favors the idea that CD9 expression does not regulate MMP expression via TIMPs.

It is well established that MMP-9 increases in cancer cell lines is a consequence of increased PI3K-Akt activity [55]–[58]. Thant, et al. found the PI3K-Akt pathway to be essential for FN-dependent MMP-9 secretion and invasion of ovarian cancer cells [59]. We have previously demonstrated in two cell lines that CD9 expression enhances Akt phosphorylation [60], [61], and our preliminary studies with CD9-HT1080 cells indicated a 25% decrease in MMP-9 gelatinolytic band intensity using the PI3K inhibitor LY294002 in a gelatin zymography analysis of CD9-HT1080 conditioned media (Herr et al., unpublished observations). We propose that the same PI3K-Akt pathway may be responsible for our observed increase in MMP-9 expression and secretion in CD9-HT1080 cells. Chen, et al. substantiate this hypothesis by demonstrating that CD9 expression in HT1080 cells resulted in increased Akt phosphorylation [24]. Future studies in our laboratory will focus on mechanisms associated with CD9 regulation of MMP-9 activity via the PI3K-Akt pathway.

The second extracellular loop (EC2) of CD9 is responsible for modulating multiple cellular processes including the upregulation of diptheria toxin binding [35], and gamete fusion [37]. Deletion of CD9 EC2 in a Chinese Hamster Ovary cell line led to decreased motility and increased cell adhesion and fibronectin matrix assembly [32], [36]. Herein, we demonstrate that CD9 EC2 was critical in eliciting MMP-9 expression in CD9-HT1080 cells. Upon EC2 deletion, the observed increases in MMP-9 expression and release were reduced to levels found in Mock-HT1080 cells or after silencing of the proteinase. Furthermore, the increase in the invasive phenotype of CD9-HT1080 cells was reduced upon deletion of CD9 EC2. These findings confirm and extend our previously published data that CD9 EC2 is of immense importance in the full functioning of this tetraspanin.

In summary, our findings demonstrate for the first time a specific link between tetraspanin CD9 and metalloproteinase MMP-9 expression that significantly affects the invasive phenotype of HT1080 cells. Therefore, CD9 consequently may play an important role in tumor cell invasion and promoting metastasis. This potential relationship between CD9 expression and the tumor cell phenotype is supported by studies that used inhibitory anti-CD9 antibodies to decrease tumor weight in two separate *in vivo* models [13], [62]. Additionally, we attribute CD9 EC2 region as being responsible and a potentially new target for regulating MMP-9 production. Together, these key findings support a model in which tetraspanin CD9 may have considerable therapeutic potential for the treatment of metastasizing cancers.

## Supporting Information

Table S1 **List of primers used for tetraspanin and integrin qRT-PCR analysis.**
(DOCX)

Table S2 **List of primers used for MMP and TIMP qRT-PCR analysis.**
(DOCX)

## References

[1] Nagase H, Woessner JF Jr (1999) Matrix metalloproteinases. J Biol Chem 274: 21491–21494. 10419448 10.1074/jbc.274.31.21491

[2] Page-McCaw A, Ewald AJ, Werb Z (2007) Matrix metalloproteinases and the regulation of tissue remodelling. Nat Rev Mol Cell Biol 8: 221–233. 17318226 10.1038/nrm2125 PMC2760082

[3] Zucker S, Cao J, Chen WT (2000) Critical appraisal of the use of matrix metalloproteinase inhibitors in cancer treatment. Oncogene 19: 6642–6650. 11426650 10.1038/sj.onc.1204097

[4] Visse R, Nagase H (2003) Matrix metalloproteinases and tissue inhibitors of metalloproteinases: structure, function, and biochemistry. Circ Res 92: 827–839. 12730128 10.1161/01.RES.0000070112.80711.3D

[5] Skiles JW, Gonnella NC, Jeng AY (2004) The design, structure, and clinical update of small molecular weight matrix metalloproteinase inhibitors. Curr Med Chem 11: 2911–2977. 15544483 10.2174/0929867043364018

[6] Close DR (2001) Matrix metalloproteinase inhibitors in rheumatic diseases. Ann Rheum Dis 60 Suppl 3iii62–67. 11890658 10.1136/ard.60.90003.iii62 PMC1766680

[7] Kanetaka K, Sakamoto M, Yamamoto Y, Takamura M, Kanematsu T, et al (2003) Possible involvement of tetraspanin CO-029 in hematogenous intrahepatic metastasis of liver cancer cells. J Gastroenterol Hepatol 18: 1309–1314. 14535989 10.1046/j.1440-1746.2003.03182.x

[8] Zijlstra A, Lewis J, Degryse B, Stuhlmann H, Quigley JP (2008) The inhibition of tumor cell intravasation and subsequent metastasis via regulation of in vivo tumor cell motility by the tetraspanin CD151. Cancer Cell 13: 221–234. 18328426 10.1016/j.ccr.2008.01.031 PMC3068919

[9] Yanez-Mo M, Barreiro O, Gordon-Alonso M, Sala-Valdes M, Sanchez-Madrid F (2009) Tetraspanin-enriched microdomains: a functional unit in cell plasma membranes. Trends Cell Biol 19: 434–446. 19709882 10.1016/j.tcb.2009.06.004

[10] Berditchevski F (2001) Complexes of tetraspanins with integrins: more than meets the eye. J Cell Sci 114: 4143–4151. 11739647 10.1242/jcs.114.23.4143

[11] Zoller M (2009) Tetraspanins: push and pull in suppressing and promoting metastasis. Nat Rev Cancer 9: 40–55. 19078974 10.1038/nrc2543

[12] Wang HX, Li Q, Sharma C, Knoblich K, Hemler ME (2011) Tetraspanin protein contributions to cancer. Biochemical Society transactions 39: 547–552. 21428937 10.1042/BST0390547

[13] Hwang JR, Jo K, Lee Y, Sung BJ, Park YW, et al (2012) Upregulation of CD9 in ovarian cancer is related to the induction of TNF-alpha gene expression and constitutive NF-kappaB activation. Carcinogenesis 33: 77–83. 22095071 10.1093/carcin/bgr257

[14] Kischel P, Bellahcene A, Deux B, Lamour V, Dobson R, et al (2012) Overexpression of CD9 in Human Breast Cancer Cells Promotes the Development of Bone Metastases. Anticancer Res 32: 5211–5220. 23225418

[15] Iwasaki T, Takeda Y, Maruyama K, Yokosaki Y, Tsujino K, et al.. (2012) Deletion of tetraspanin CD9 diminishes lymphangiogenesis in vivo and in vitro. J Biol Chem. 10.1074/jbc.M112.424291 PMC3554885 23223239

[16] Hori H, Yano S, Koufuji K, Takeda J, Shirouzu K (2004) CD9 expression in gastric cancer and its significance. The Journal of surgical research 117: 208–215. 15047125 10.1016/j.jss.2004.01.014

[17] Soyuer S, Soyuer I, Unal D, Ucar K, Yildiz OG, et al (2010) Prognostic significance of CD9 expression in locally advanced gastric cancer treated with surgery and adjuvant chemoradiotherapy. Pathology, research and practice 206: 607–610. 10.1016/j.prp.2010.04.004 20547009

[18] Wang JC, Begin LR, Berube NG, Chevalier S, Aprikian AG, et al (2007) Down-regulation of CD9 expression during prostate carcinoma progression is associated with CD9 mRNA modifications. Clinical cancer research: an official journal of the American Association for Cancer Research 13: 2354–2361. 17406028 10.1158/1078-0432.CCR-06-1692

[19] Takino T, Miyamori H, Kawaguchi N, Uekita T, Seiki M, et al (2003) Tetraspanin CD63 promotes targeting and lysosomal proteolysis of membrane-type 1 matrix metalloproteinase. Biochem Biophys Res Commun 304: 160–166. 12705901 10.1016/s0006-291x(03)00544-8

[20] Yanez-Mo M, Barreiro O, Gonzalo P, Batista A, Megias D, et al (2008) MT1-MMP collagenolytic activity is regulated through association with tetraspanin CD151 in primary endothelial cells. Blood 112: 3217–3226. 18663148 10.1182/blood-2008-02-139394

[21] Jung KK, Liu XW, Chirco R, Fridman R, Kim HR (2006) Identification of CD63 as a tissue inhibitor of metalloproteinase-1 interacting cell surface protein. EMBO J 25: 3934–3942. 16917503 10.1038/sj.emboj.7601281 PMC1560352

[22] Takeda Y, He P, Tachibana I, Zhou B, Miyado K, et al (2008) Double deficiency of tetraspanins CD9 and CD81 alters cell motility and protease production of macrophages and causes chronic obstructive pulmonary disease-like phenotype in mice. J Biol Chem 283: 26089–26097. 18662991 10.1074/jbc.M801902200 PMC3258854

[23] Fujita Y, Shiomi T, Yanagimoto S, Matsumoto H, Toyama Y, et al (2006) Tetraspanin CD151 is expressed in osteoarthritic cartilage and is involved in pericellular activation of pro-matrix metalloproteinase 7 in osteoarthritic chondrocytes. Arthritis Rheum 54: 3233–3243. 17009258 10.1002/art.22140

[24] Chen S, Sun Y, Jin Z, Jing X (2011) Functional and biochemical studies of CD9 in fibrosarcoma cell line. Molecular and cellular biochemistry 350: 89–99. 21161334 10.1007/s11010-010-0685-1

[25] Funakoshi T, Tachibana I, Hoshida Y, Kimura H, Takeda Y, et al (2003) Expression of tetraspanins in human lung cancer cells: frequent downregulation of CD9 and its contribution to cell motility in small cell lung cancer. Oncogene 22: 674–687. 12569360 10.1038/sj.onc.1206106

[26] Murayama Y, Shinomura Y, Oritani K, Miyagawa J, Yoshida H, et al (2008) The tetraspanin CD9 modulates epidermal growth factor receptor signaling in cancer cells. J Cell Physiol 216: 135–143. 18247373 10.1002/jcp.21384

[27] Hanyu A, Kojima K, Hatake K, Nomura K, Murayama H, et al (2009) Functional in vivo optical imaging of tumor angiogenesis, growth, and metastasis prevented by administration of anti-human VEGF antibody in xenograft model of human fibrosarcoma HT1080 cells. Cancer Sci 100: 2085–2092. 19719773 10.1111/j.1349-7006.2009.01305.x PMC11158413

[28] Zhang C, Li Y, Shi X, Kim SK (2010) Inhibition of the expression on MMP-2, 9 and morphological changes via human fibrosarcoma cell line by 6,6′-bieckol from marine alga Ecklonia cava. BMB Rep 43: 62–68. 20132738 10.5483/bmbrep.2010.43.1.062

[29] Baldassarre M, Razinia Z, Brahme NN, Buccione R, Calderwood DA (2012) Filamin A controls matrix metalloproteinase activity and regulates cell invasion in human fibrosarcoma cells. J Cell Sci 125: 3858–3869. 22595522 10.1242/jcs.104018 PMC3462082

[30] Peterfia B, Fule T, Baghy K, Szabadkai K, Fullar A, et al (2012) Syndecan-1 enhances proliferation, migration and metastasis of HT-1080 cells in cooperation with syndecan-2. PLoS One 7: e39474. 22745764 10.1371/journal.pone.0039474 PMC3383727

[31] Jennings LK, Fox CF, Kouns WC, McKay CP, Ballou LR, et al (1990) The activation of human platelets mediated by anti-human platelet p24/CD9 monoclonal antibodies. J Biol Chem 265: 3815–3822. 2303480

[32] Cook GA, Longhurst CM, Grgurevich S, Cholera S, Crossno JT Jr, et al (2002) Identification of CD9 extracellular domains important in regulation of CHO cell adhesion to fibronectin and fibronectin pericellular matrix assembly. Blood 100: 4502–4511. 12453879 10.1182/blood.V100.13.4502

[33] Tohami T, Drucker L, Shapiro H, Radnay J, Lishner M (2007) Overexpression of tetraspanins affects multiple myeloma cell survival and invasive potential. FASEB J 21: 691–699. 17210782 10.1096/fj.06-6610com

[34] Hong IK, Jin YJ, Byun HJ, Jeoung DI, Kim YM, et al (2006) Homophilic interactions of Tetraspanin CD151 up-regulate motility and matrix metalloproteinase-9 expression of human melanoma cells through adhesion-dependent c-Jun activation signaling pathways. J Biol Chem 281: 24279–24292. 16798740 10.1074/jbc.M601209200

[35] Hasuwa H, Shishido Y, Yamazaki A, Kobayashi T, Yu X, et al (2001) CD9 amino acids critical for upregulation of diphtheria toxin binding. Biochem Biophys Res Commun 289: 782–790. 11735113 10.1006/bbrc.2001.6053

[36] Longhurst CM, Jacobs JD, White MM, Crossno JT Jr, Fitzgerald DA, et al (2002) Chinese hamster ovary cell motility to fibronectin is modulated by the second extracellular loop of CD9. Identification of a putative fibronectin binding site. J Biol Chem 277: 32445–32452. 12068019 10.1074/jbc.M204420200

[37] Zhu GZ, Miller BJ, Boucheix C, Rubinstein E, Liu CC, et al (2002) Residues SFQ (173–175) in the large extracellular loop of CD9 are required for gamete fusion. Development 129: 1995–2002. 11934865 10.1242/dev.129.8.1995

[38] Ovalle S, Gutierrez-Lopez MD, Olmo N, Turnay J, Lizarbe MA, et al (2007) The tetraspanin CD9 inhibits the proliferation and tumorigenicity of human colon carcinoma cells. Int J Cancer 121: 2140–2152. 17582603 10.1002/ijc.22902

[39] Furuya M, Kato H, Nishimura N, Ishiwata I, Ikeda H, et al (2005) Down-regulation of CD9 in human ovarian carcinoma cell might contribute to peritoneal dissemination: morphologic alteration and reduced expression of beta1 integrin subsets. Cancer Res 65: 2617–2625. 15805258 10.1158/0008-5472.CAN-04-3123

[40] Saito Y, Tachibana I, Takeda Y, Yamane H, He P, et al (2006) Absence of CD9 enhances adhesion-dependent morphologic differentiation, survival, and matrix metalloproteinase-2 production in small cell lung cancer cells. Cancer Res 66: 9557–9565. 17018612 10.1158/0008-5472.CAN-06-1131

[41] Sordat I, Decraene C, Silvestre T, Petermann O, Auffray C, et al (2002) Complementary DNA arrays identify CD63 tetraspanin and alpha3 integrin chain as differentially expressed in low and high metastatic human colon carcinoma cells. Lab Invest 82: 1715–1724. 12480921 10.1097/01.lab.0000044350.18215.0d

[42] Lafleur MA, Xu D, Hemler ME (2009) Tetraspanin proteins regulate membrane type-1 matrix metalloproteinase-dependent pericellular proteolysis. Mol Biol Cell 20: 2030–2040. 19211836 10.1091/mbc.E08-11-1149 PMC2663921

[43] Testa JE, Brooks PC, Lin JM, Quigley JP (1999) Eukaryotic expression cloning with an antimetastatic monoclonal antibody identifies a tetraspanin (PETA-3/CD151) as an effector of human tumor cell migration and metastasis. Cancer Res 59: 3812–3820. 10447000

[44] Kohno M, Hasegawa H, Miyake M, Yamamoto T, Fujita S (2002) CD151 enhances cell motility and metastasis of cancer cells in the presence of focal adhesion kinase. Int J Cancer 97: 336–343. 11774285 10.1002/ijc.1605

[45] Zoller M (2006) Gastrointestinal tumors: metastasis and tetraspanins. Z Gastroenterol 44: 573–586. 16823699 10.1055/s-2006-926795

[46] Riikonen T, Westermarck J, Koivisto L, Broberg A, Kahari VM, et al (1995) Integrin alpha 2 beta 1 is a positive regulator of collagenase (MMP-1) and collagen alpha 1(I) gene expression. The Journal of biological chemistry 270: 13548–13552. 7768957 10.1074/jbc.270.22.13548

[47] Pilcher BK, Dumin JA, Sudbeck BD, Krane SM, Welgus HG, et al (1997) The activity of collagenase-1 is required for keratinocyte migration on a type I collagen matrix. The Journal of cell biology 137: 1445–1457. 9182674 10.1083/jcb.137.6.1445 PMC2132537

[48] Nakazawa Y, Sato S, Naito M, Kato Y, Mishima K, et al (2008) Tetraspanin family member CD9 inhibits Aggrus/podoplanin-induced platelet aggregation and suppresses pulmonary metastasis. Blood 112: 1730–1739. 18541721 10.1182/blood-2007-11-124693

[49] Zvieriev V, Wang JC, Chevrette M (2005) Over-expression of CD9 does not affect in vivo tumorigenic or metastatic properties of human prostate cancer cells. Biochemical and Biophysical Research Communications 337: 498–504. 16198313 10.1016/j.bbrc.2005.09.073

[50] Zhu X, Tai W, Shi W, Song Y, Zhang H, et al (2012) Matrix metalloproteinase-9 silencing by RNA interference promotes the adhesive-invasive switch in HT1080 human fibrosarcoma cells. Clin Lab 58: 313–322. 22582506

[51] Jee BK, Park KM, Surendran S, Lee WK, Han CW, et al (2006) KAI1/CD82 suppresses tumor invasion by MMP9 inactivation via TIMP1 up-regulation in the H1299 human lung carcinoma cell line. Biochemical and biophysical research communications 342: 655–661. 16488391 10.1016/j.bbrc.2006.01.153

[52] Okada Y, Gonoji Y, Naka K, Tomita K, Nakanishi I, et al (1992) Matrix metalloproteinase 9 (92-kDa gelatinase/type IV collagenase) from HT 1080 human fibrosarcoma cells. Purification and activation of the precursor and enzymic properties. J Biol Chem 267: 21712–21719. 1400481

[53] Leco KJ, Apte SS, Taniguchi GT, Hawkes SP, Khokha R, et al (1997) Murine tissue inhibitor of metalloproteinases-4 (Timp-4): cDNA isolation and expression in adult mouse tissues. FEBS Lett 401: 213–217. 9013889 10.1016/s0014-5793(96)01474-3

[54] Greene J, Wang M, Liu YE, Raymond LA, Rosen C, et al (1996) Molecular cloning and characterization of human tissue inhibitor of metalloproteinase 4. J Biol Chem 271: 30375–30380. 8939999 10.1074/jbc.271.48.30375

[55] Cheng JC, Chou CH, Kuo ML, Hsieh CY (2006) Radiation-enhanced hepatocellular carcinoma cell invasion with MMP-9 expression through PI3K/Akt/NF-kappaB signal transduction pathway. Oncogene 25: 7009–7018. 16732316 10.1038/sj.onc.1209706

[56] Cho SJ, Chae MJ, Shin BK, Kim HK, Kim A (2008) Akt- and MAPK-mediated activation and secretion of MMP-9 into stroma in breast cancer cells upon heregulin treatment. Mol Med Report 1: 83–88. 21479382

[57] Chen JS, Wang Q, Fu XH, Huang XH, Chen XL, et al (2009) Involvement of PI3K/PTEN/AKT/mTOR pathway in invasion and metastasis in hepatocellular carcinoma: Association with MMP-9. Hepatol Res 39: 177–186. 19208038 10.1111/j.1872-034X.2008.00449.x

[58] Zhu X, Wang L, Zhang B, Li J, Dou X, et al (2011) TGF-beta1-induced PI3K/Akt/NF-kappaB/MMP9 signalling pathway is activated in Philadelphia chromosome-positive chronic myeloid leukaemia hemangioblasts. J Biochem 149: 405–414. 21288887 10.1093/jb/mvr016

[59] Thant AA, Nawa A, Kikkawa F, Ichigotani Y, Zhang Y, et al (2000) Fibronectin activates matrix metalloproteinase-9 secretion via the MEK1-MAPK and the PI3K-Akt pathways in ovarian cancer cells. Clin Exp Metastasis 18: 423–428. 11467775 10.1023/a:1010921730952

[60] Kotha J, Longhurst C, Appling W, Jennings LK (2008) Tetraspanin CD9 regulates beta 1 integrin activation and enhances cell motility to fibronectin via a PI-3 kinase-dependent pathway. Exp Cell Res 314: 1811–1822. 18358474 10.1016/j.yexcr.2008.01.024

[61] Kotha J, Zhang C, Longhurst CM, Lu Y, Jacobs J, et al (2009) Functional relevance of tetraspanin CD9 in vascular smooth muscle cell injury phenotypes: a novel target for the prevention of neointimal hyperplasia. Atherosclerosis 203: 377–386. 18799160 10.1016/j.atherosclerosis.2008.07.036

[62] Nakamoto T, Murayama Y, Oritani K, Boucheix C, Rubinstein E, et al (2009) A novel therapeutic strategy with anti-CD9 antibody in gastric cancers. Journal of Gastroenterology 44: 889–896. 19468669 10.1007/s00535-009-0081-3

