# Supplementary material for: Tetraspanin CD9 Promotes the Invasive Phenotype of Human Fibrosarcoma Cells via Upregulation of Matrix Metalloproteinase-9
Source: PLoS One. 2013 Jun 28;8(6):e67766. doi: 10.1371/journal.pone.0067766 (PMC3696041; doi:10.1371/journal.pone.0067766)
Supplement: Table S1 — List of primers used for tetraspanin and integrin qRT-PCR analysis. (DOCX) [file pone.0067766.s001.docx]

**Table S1.** List of primers used for tetraspanin and integrin qRT-PCR analysis.

| **Gene** | **Forward Primer** | **Reverse Primer** |
| --- | --- | --- |
| CD9 TM1 | GAGGCACCAAGTGCATCAA | AGCCATAGTCCAATGGCAAG |
| CD9 EC2 | GGCGTGGAACAGTTTATCTCA | GATGGCATCAGGACAGGACT |
| CD63 | TGAAATGTGTGAAGTTCTTGCTC | CAATCAGTCCCACTGCACA |
| CD81 | TCGTCTTCAATTTCGTCTTCTG | CTCCCAGCTCCAGATACAGG |
| CD151 | GGAGCTTCTGTCCACCTGTC | TCGTTGAACTCACCCATCCT |
| α2 | TCGTGCACAGTTTTGAAGATG | TGGAACACTTCCTGTTGTTACC |
| α4 | GATGAAAATGAGCCTGAAACG | GCCATACTATTGCCAGTGTTGA |
| α5 | ACCCTGCCGCTCAGATTT | AAAACCACACGGCCAGTC |
| α6 | TGGCCTCTTCATTTGGCTAT | AAAATACTGTGGGGCTCCAAT |
| β1 | TTCGATGCCATCATGCAA | ACACCAGCAGCCGTGTAAC |
