# Supplementary material for: Tetraspanin CD9 Promotes the Invasive Phenotype of Human Fibrosarcoma Cells via Upregulation of Matrix Metalloproteinase-9
Source: PLoS One. 2013 Jun 28;8(6):e67766. doi: 10.1371/journal.pone.0067766 (PMC3696041; doi:10.1371/journal.pone.0067766)
Supplement: Table S2 — List of primers used for MMP and TIMP qRT-PCR analysis. (DOCX) [file pone.0067766.s002.docx]

**Table S2.** List of primers used for MMP and TIMP qRT-PCR analysis.

| **Gene** | **Forward Primer** | **Reverse Primer** |
| --- | --- | --- |
| MMP-1 | GCTAACCTTTGATGCTATAACTACGA | TTTGTGCGCATGTAGAATCTG |
| MMP-2 | ATAACCTGGATGCCGTCGT | AGGCACCCTTGAAGAAGTAGC |
| MMP-3 | GCAAGGACCTCGTTTTCATT | CTCTTGGGTATCCAGCTCGT |
| MMP-7 | TGGACGGATGGTAGCAGTCT | TCTCCATTTCCATAGGTTGGAT |
| MMP-8 | GGGAACGCACTAACTTGACC | TTCAAAGGCATCCTTGATAGC |
| MMP-9 | GAACCAATCTCACCGACAGG | GCCACCCGAGTGTAACCATA |
| MMP-14 | CTGTCAGGAATGAGGATCTGAA | AGGGGTCACTGGAATGCTC |
| MMP-16 | AGGGCATCCAGAAGATATATGG | GGCACTGTCGGTAGAGGTCTT |
| TIMP-1 | GGGCTTCACCAAGACCTACA | TGCAGGGGATGGATAAACAG |
| TIMP-2 | GAAGAGCTTGAACCAACAGGT | CGGGGAGGAGATGTAGCAC |
| TIMP-3 | TGCAACTCCGACATCGTG | AAGGGCCCCTCCTTTACC |
